# Development and Validation of a Predictive Model of Hypovitaminosis D in General Adult Population: SCOPYD Study

**DOI:** 10.3390/nu13082526

**Published:** 2021-07-23

**Authors:** Marie Viprey, Blandine Merle, Benjamin Riche, Julie Freyssenge, Pascal Rippert, Mohammed-Amine Chakir, Thierry Thomas, Sandrine Malochet-Guinamand, Bernard Cortet, Véronique Breuil, Roland Chapurlat, Marie-Hélène Lafage Proust, Marie-Christine Carlier, Jean-Baptiste Fassier, Julie Haesebaert, Pascal Caillet, Muriel Rabilloud, Anne-Marie Schott

**Affiliations:** 1 Research on Healthcare Performance RESHAPE, INSERM U1290, Université Claude Bernard Lyon 1, 69003 Lyon, France; j.freyssenge@resuval.fr (J.F.); julie.haesebaert01@chu-lyon.fr (J.H.); anne-marie.schott-pethelaz@chu-lyon.fr (A.-M.S.); 2 Hospices Civils de Lyon, Pôle de Sante Publique, 69003 Lyon, France; pascal.rippert@chu-lyon.fr (P.R.); chakirmedamine@gmail.com (M.-A.C.); jean-baptiste.fassier@chu-lyon.fr (J.-B.F.); 3 INSERM UMR 1033, Université de Lyon, 69000 Lyon, France; blandine.merle@inserm.fr (B.M.); roland.chapurlat@chu-lyon.fr (R.C.); 4 Université Lyon 1, Université de Lyon, 69000 Lyon, France; benjamin.riche@chu-lyon.fr (B.R.); muriel.rabilloud@chu-lyon.fr (M.R.); 5 Hospices Civils de Lyon, Pôle Santé Publique, Service de Biostatistique et Bioinformatique, 69003 Lyon, France; 6 CNRS, UMR 5558, Laboratoire de Biométrie et Biologie Évolutive, Équipe Biostatistique-Santé, 69100 Villeurbanne, France; 7 RESCUe-RESUVal Network, Lucien Hussel Hospital, 38200 Vienne, France; 8 CHU Saint-Etienne, Hôpital Nord, Service de Rhumatologie, INSERM U1059, Laboratoire de Biologie intégrée du Tissu Osseux, Université de Lyon, 42000 Saint-Etienne, France; thierry.thomas@chu-st-etienne.fr (T.T.); lafagemh@univ-st-etienne.fr (M.-H.L.P.); 9 CHU Clermont-Ferrand, Hôpital Gabriel Montpied, Service de Rhumatologie, 63000 Clermont-Ferrand, France; smalochet@chu-clermontferrand.fr; 10 CHRU de Lille, Service de Rhumatologie, 59000 Lille, France; bernard.cortet@chru-lille.fr; 11 CHU de Nice, Service de Rhumatologie, 06000 Nice, France; breuil.v@chu-nice.fr; 12 Hospices Civils de Lyon, Hôpital Edouard Herriot, Service de Rhumatologie, 69003 Lyon, France; 13 Département de Biologie, Centre Hospitalier Lyon-Sud, 69495 Pierre-Bénite, France; mccarlier@gmail.com; 14 Unité Mixte de Recherche Epidémiologique et de Surveillance Transport Travail Environnement (UMRESTTE) UMR T9405, Université Claude Bernard Lyon 1, 69000 Lyon, France; 15 CHU de Nantes, Unité de Santé Publique Interventionnelle, 44000 Nantes, France; pascal.caillet@chu-nantes.fr

**Keywords:** vitamin D deficiency, severe vitamin D deficiency, hypovitaminosis D, predictive model, adult, sun exposure assessment

## Abstract

The worldwide global increase in serum 25-hydroxyvitamin D (25(OH)D) measurements has led some countries to restrict reimbursement for certain clinical situations only. Another approach could consist in providing physicians with screening tools in order to better target blood test prescription. The objective of the SCOPYD study was to identify the best combination of predictors of serum VitD concentration among adults aged 18–70 years. Potential risk factors for VitD deficiency were collected using a comprehensive self-administered questionnaire. A multivariable linear regression was used to build a predictive model of serum 25(OH)D concentration. Among 2488 participants, 1080 (43.4%) had VitD deficiency (<50 nmol/L) and 195 (7.8%) had severe deficiency (<25 nmol/L). The final model included sunlight exposure in the preceding week and during the last holidays, month of blood sampling, age, sex, body mass index, skin phototype, employment, smoking, sport practice, latitude, and VitD supplementation in preceding year. The area under the curve was 0.82 (95% CI (0.78; 0.85)) for severe deficiency. The model predicted severe deficiency with a sensitivity of 77.9% (95% CI (69.1; 85.7)) and a specificity of 68.3% (95% CI (64.8; 71.9)). We identified a set of predictors of severe VitD deficiency that are easy to collect in routine that may help to better target patients for serum 25(OH)D concentration determination.

## 1. Introduction

Vitamin D deficiency has long been recognized as a cause of osteomalacia in adults and rickets in children. A low vitamin D concentration is also considered as a risk factor for bone fragility [1]. In the last decades, many observational studies have found that serum vitamin D concentration was inversely correlated with the occurrence of multiple chronic disorders (e.g., cancer, cardiovascular disease, diabetes, and autoimmune disorders). Due to this growing interest in the pleiotropic putative effects of vitamin D, a massive increase in the number of serum 25-hydroxyvitamin D (25(OH)D) measurements has been observed worldwide [2,3,4,5,6]. In this context, providing physicians with clinical tools to identify and discriminate between high-risk patients and those presenting a very low risk of vitamin D deficiency should help them to better target the patients for whom vitamin D concentration determination is indicated.

A large number of studies have been conducted to identify the risk factors of vitamin D deficiency and try to develop clinical scores. However, most studies have addressed this question only in specific sub-groups of the population such as post-menopausal women [7,8,9,10], older adults [11,12,13], or pregnant women [14], in restricted geographical areas [15,16,17], or without considering critical risk factors such as sun exposure [18,19] or other known factors of hypovitaminosis D [15,20,21]. Furthermore, studies that have addressed individual sun exposure have measured it incompletely or without precision regarding the surface exposed. Consequently, there is a need for further studies in the general population to address more comprehensively all the risk factors (and more precisely individual sun exposure) associated with vitamin D deficiency in order to further develop a diagnostic tool based on risk factors that would be easily assessed through a self-administered questionnaire.

The objective of the present study was to identify the best combination of factors to predict the concentration of serum vitamin D among adults aged 18 to 70 years drawn from the general population.

## 2. Materials and Methods

### 2.1. Study Design and Participants

A multicenter national cross-sectional study was performed. We used the transparent reporting of a multivariable prediction model for individual prognosis or diagnosis (TRIPOD) [22] to guide the reporting.

Outpatients were recruited from rheumatology, dermatology, anesthesiology, occupational medicine, and sports medicine departments in 5 university hospitals (Lyon, Clermont-Ferrand, Saint Etienne, Nice, Lille) representing various geographic regions of France to take into account the vitamin D variability associated with latitude (43° to 50°). The inclusion period stretched over a whole year to take into account the seasonal variability of vitamin D concentration, and it eventually lasted 14 months (from September 2016 to November 2017) to meet the initial sample size calculation. Men and women aged 18 to 70 years old were included. Exclusion criteria were health disorders that could possibly impact the vitamin D status such as renal failure (severe renal impairment, dialysis, and kidney transplant), known hepatic impairment, gastrointestinal disorders (celiac disease, Crohn’s disease, ulcerative colitis, bariatric surgery, and gastrointestinal surgery with stoma), known primary hypo/hyperparathyroidism, and bone cancer/metastases current or within the last 2 years. Other exclusion criteria were current or recent (less than one year) participation in a study related to vitamin D, pregnancy, or breast-feeding, ongoing antiepileptic or antiretroviral treatment, ongoing long-term glucocorticoid treatment (>3 months), legal incapacity or limited legal capacity, no affiliation to the national French health insurance, or having received at least 80,000 IU vitamin D in the last 3 months in a single dose. Conversely, patients could be currently treated with low daily doses of vitamin D or could have received higher unique dose of vitamin D more than 3 months before the inclusion in the study.

### 2.2. Outcome

The primary objective was to identify the best combination of factors to predict the concentration of serum vitamin D, and the corresponding primary outcome was the circulating 25(OH)D concentration as a continuous variable and measured as described below. The secondary objective was to evaluate the performance of the model to identify people having a vitamin D deficiency and those having a severe vitamin D deficiency. The gold standard used to measure the sensitivity and specificity of the models was based on the result of the serum 25(OH)D measurements. A vitamin D deficiency was defined as a serum 25(OH)D concentration <50 nmol/L [23,24], and a severe vitamin D deficiency was defined as a serum 25(OH)D measurement <25 nmol/L [25].

### 2.3. Serum 25(OH)D Measurement

Blood was collected from all participants on the day of questionnaire completion, serum was separated and kept at −80 °C until assayed. Serum 25(OH)D concentration was determined by chemiluminescent immunoassay technology (Liaison XL^®^, DiaSorin, Saluggia, Italy) in duplicate. Measurements were performed blinded from the questionnaires results, in one of the three participating centers in Lyon (77%), Nice (14%), and Clermont-Ferrand (9%). The laboratories of these centers are affiliated to an external quality control program allowing inter-center standardization. The intra- and inter-assay coefficients of variation were <10%.

### 2.4. Risk Factors

For collecting data assessing potential risk factors for vitamin deficiency, a self-assessment questionnaire was developed in four steps. The first step was to gather all relevant information through a literature review of factors potentially associated with vitamin D concentration or hypovitaminosis D symptoms in order to build a template for the questionnaire. The second step was the validation of the questionnaire content by a multidisciplinary group of rheumatologists, biologists, and public health specialists. The third step was a test of the first version on 13 outpatients for evaluating the study feasibility, understandability, and the appropriateness of the questionnaire, and for changing its content accordingly. The fourth step was a test of the second version in real study conditions on 19 outpatients, which led to the final version after minor changes.

The self-administered questionnaire was provided to each participant upon arrival in the ward. A large number of potential risk factors for vitamin D deficiency were collected through this questionnaire, which contained six sections (Appendix A).

The first section was related to socio-economic characteristics (age, sex, country of birth, education level, employment status, and place of residence). The latitude of the place of residence was classified in three areas according to the French Lambert zone projection system [26]: North for latitudes of 48°15′ or above, Center for latitudes between 44°45′ and 48°14′, and South/Corsica for latitudes between 42°76′ and 44°44′.

The second section was composed of clinical characteristics (weight, height, skin phototype, current smoking status (Yes/No), physical activities, chronic muscle, joint, or bone pain with no known cause, and for women number of pregnancies, age at first pregnancy, year of birth of last child, menopausal status). Regarding the skin phototype, participants were asked to choose one of the 6 categories of the Fitzpatrick scale [27]. Then, they were grouped into 3 categories, (1) light-colored skin (type I to III), (2) tanned skin (type IV), and (3) dark skin (type V and VI). Physical activities were classified in 3 categories according to their intensity based on the Mitchell classification [28]. This classification has two dimensions (static and dynamic component of the activity), if the activity was rated as low on one dimension and low or moderate on the other, it was classified as “low-intensity sports”; if the activity was rated as moderate on both dimensions, it was classified as “medium-intensity sports”; if the activity was rated as high on at least one dimension, it was classified as “high-intensity sport”.

The third section included data on sun exposure. All situations of sun exposure were collected: usual sun exposure during work time, usual sun exposure during leisure time, recent exposure over the 7 days prior to the blood test, and sun exposure during holidays over the last 12 months. Regarding this last question, a “significant sun exposure” was defined as having exposed one’s bust during at least one period of holidays over the last 12 months (Yes/No).

The fourth section contained information regarding treatments: vitamin D prescribed by physicians, over-the-counter vitamin D supplements, non-steroidal anti-inflammatory drugs, diuretics, oral contraceptives, hormone replacement therapy, and other treatments regularly taken that may influence vitamin D concentration.

The fifth section was composed of dietary sources of vitamin D (food naturally rich in vitamin D or that contains added vitamin D), and the sixth section collected data regarding exposure to artificial ultraviolet radiations.

### 2.5. Statistical Analysis

#### 2.5.1. Sample Size

The number of participants to be included in the sample used to construct the multiple linear model was determined so that the relative reduction of the model predictive ability for new participants, measured by the coefficient of determination (R-squared or R^2^), would not be more than 2.5%. Previously published predictive models had a R^2^ between 0.13 and 0.42 [7,11,18,29,30]. We hypothesized that the predictive ability of the model would be at least 0.4. For a number of 10 predictors included in the model, and a relative difference between the R^2^ and the corrected R^2^ of 2.5% (from 0.4 to 0.39), the number of participants to be included was about 1300 [31,32]. Thus, we planned to include 2500 participants, so that the model could be constructed using a randomly selected sample of 1300 participants among the 2500 and validated on the remaining 1200 participants. The model was finally constructed using all the included participants and validated using a bootstrap method as proposed by Steyerberg, who has shown that this approach is better than splitting the dataset into a training sample and a test sample [33].

#### 2.5.2. Statistical Analysis

The description of the population was carried out using the mean (±standard deviation, SD) or the median (range) for quantitative characteristics, and absolute and relative frequencies for qualitative characteristics.

A linear regression model was used to build the predictive model with the value of vitamin D measurement as the dependent variable (primary outcome). A first model was built and included the month of blood sampling modeled using a cyclic cubic spline in order to take into account the seasonality of the vitamin D measurement. The corrected R^2^ obtained using a bootstrap method was estimated to quantify the predictive ability of the model. The following step was to build bivariable models by adding separately each selected variable to the first model. For each bivariable model, the gain of predictive ability was quantified by the increase of the corrected R^2^ compared to the first model. The studied variables were selected a priori (before the statistical analysis and after questionnaire completion) so that they were fairly representative of the first five sections of the questionnaire and had a limited number of missing values. Variables of the sixth section corresponding to exposure to artificial ultraviolet radiation were not selected due to the very low number of concerned participants. The variables included in the multivariable model in addition to the month of blood sampling were those that increased significantly the model predictive ability according to the likelihood ratio test and were easy to collect by auto-questionnaire. The predictive ability of the final model was quantified by the corrected R^2^. The analysis was carried out on the complete dataset; no method of imputation was used to replace missing data.

The overall performance of the model for identifying participants with a vitamin D deficiency or a severe vitamin D deficiency was quantified by the area under the ROC curve (AUC). The sensitivity and specificity of the model for the diagnosis of vitamin D deficiency, and for the diagnosis of severe vitamin D deficiency, were estimated using a threshold corresponding to a value predicted by the model of 50 nmol/L (test positive when the predicted value ≤ 50 nmol/L).

All parameters were estimated and expressed with their 95% confidence interval (CI) using a bootstrap method. Analyses were performed using the software SAS^®^ version 9.3, and R, version 3.4 for some graphs.

## 3. Results

### 3.1. Population Characteristics and Vitamin D Concentrations

**Figure 1:**
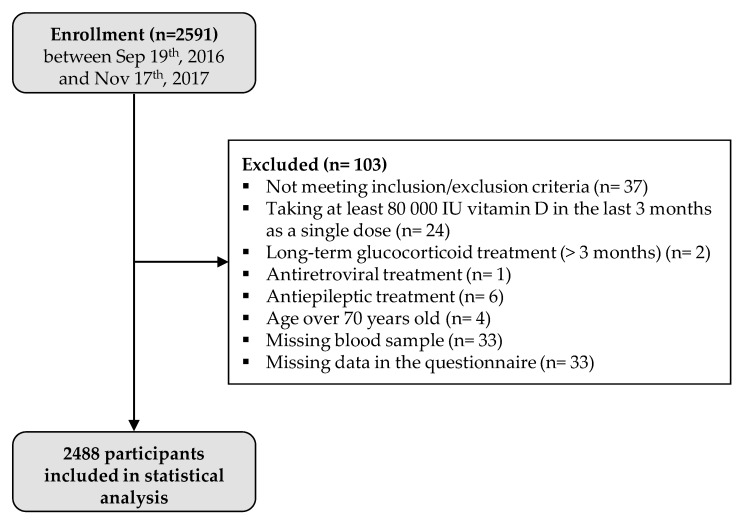
Study flow chart.

A total of 2591 participants were enrolled in the study. Serum 25(OH)D concentration was determined for 2558 individuals (33 missing blood samples), 33 (1.3%) did not fill out the questionnaire, and 37 (1.4%) were excluded because they met an exclusion criterion. Therefore, a total of 2488 participants were included in the analysis (Figure 1). Among the 2488 participants, 1513 (60.8%) were female, and information on body mass index (BMI) was available for 2473 participants: 674/2473 (27.3%) were considered as overweight (25 ≤ BMI < 30), and 418/2473 (16.9%) were considered as obese (BMI ≥ 30). Regarding skin phenotype, 1631/2475 (65.9%) participants had a light-colored skin (type I to III). A total of 525 (21.1%) participants took at least one vitamin D-supplemented product in the last 12 months, and 321 (12.9%) had had a vitamin D supplementation in the last 12 months (regular treatments with tablets or singles doses of at least 80,000 IU taken more than 3 months prior to study entry). A total of 461/2474 (18.6%) responders reported smoking, 1266 (50.9%) reported practicing sports, and 958 (38.5%) reported a “significant sun exposure” during holidays over the last 12 months (Table 1).

Serum 25(OH)D concentrations <50 nmol/L, and <25 nmol/L were found in 1080/2488 (43.4%) and 195/2488 (7.8%) participants, respectively. A sufficient vitamin D status (defined as serum 25(OH)D concentration ≥ 75 nmol/L) was found in 437/2448 (17.6%) participants.

### 3.2. Modeling of Seasonal Changes

**Figure 2:**
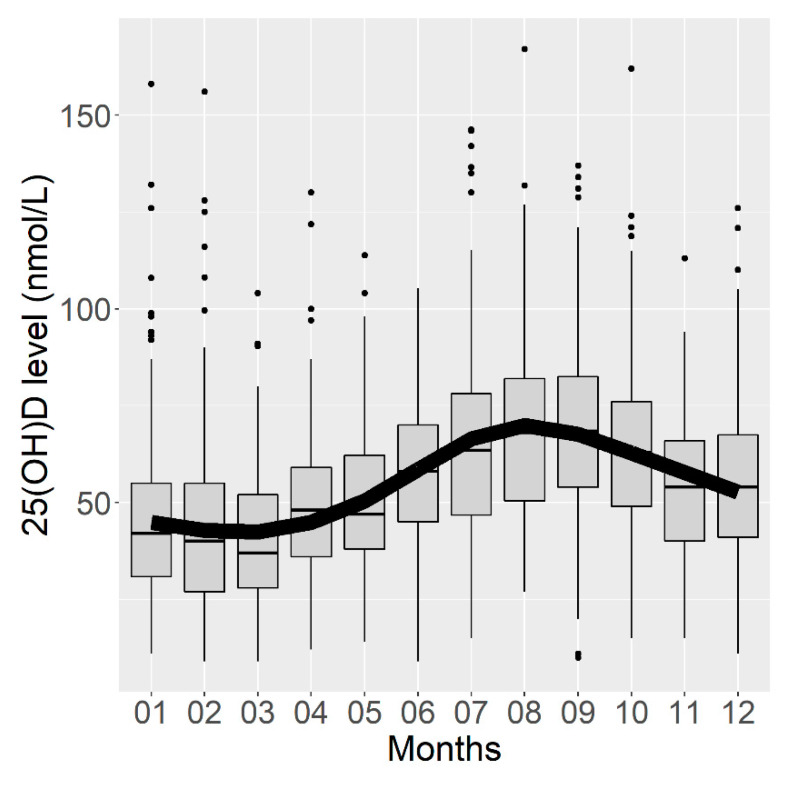
Box plots representing serum vitamin D concentrations according to the month of blood sampling and curve of vitamin D concentrations predicted by a linear regression model using a cyclic cubic spline.

The analysis of serum 25(OH)D concentrations by month of blood sampling confirmed the seasonality of the vitamin D concentration, which was the highest in August and September and the lowest concentration in February and March (Figure 2). The corrected R^2^ of this first model was estimated at 0.161 (95% CI (0.122; 0.192)), meaning that 16.1% of the variance in the vitamin D concentration could be explained by the month of blood sampling.

### 3.3. Prediction Model

The proportion of the explained variance in the vitamin D concentration increased from 0.1% to 4.2% depending on which variable was included in the bivariable model; this increase was maximal when the variable “Significant exposure during holidays over the last 12 months” was included in the bivariable model (+4.2%, corrected R^2^ = 0.203, 95% CI (0.163; 0.243); Table 2). All variables were significantly associated with the vitamin D concentration except “Intake of at least one vitamin D supplemented product in the last 12 months”.

**Figure 3:**
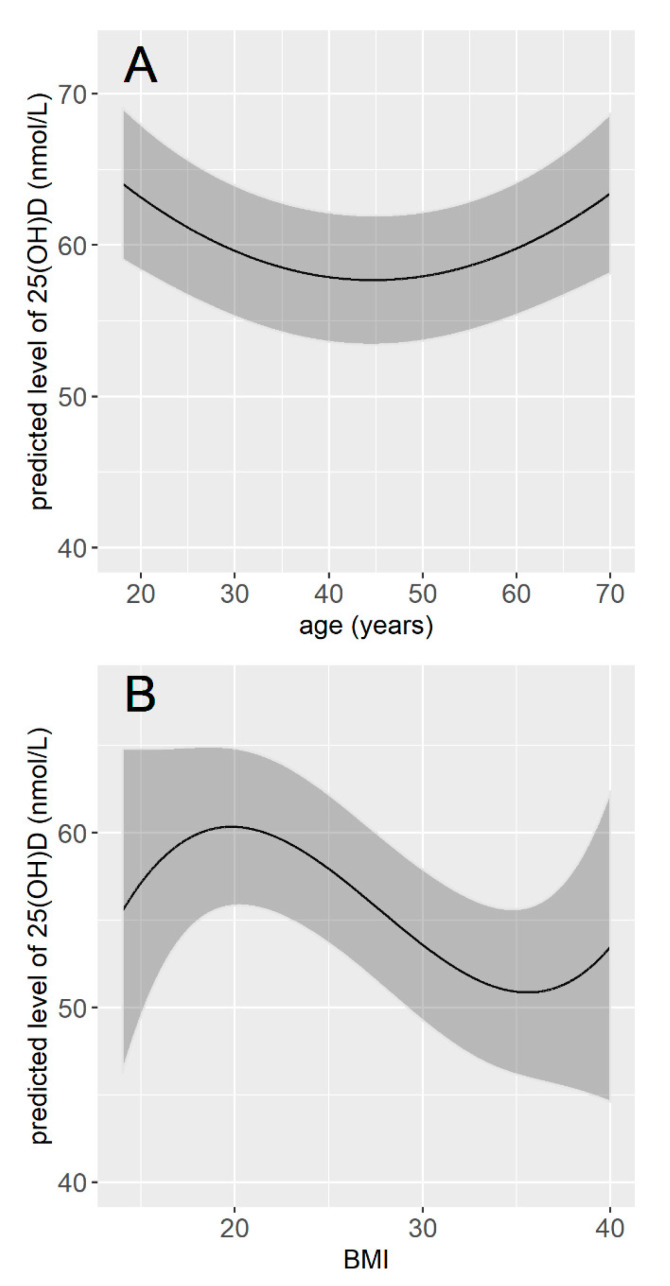
Predicted concentration of vitamin D according to age (**A**) and according to BMI (**B**). The concentrations of vitamin D were predicted for the month of January, for a male, with light-colored skin, employment, living in the North of France, non-smoker, with vitamin D supplementation in the last 12 months, a significant sun exposure over the last 12 months, an average exposure time of 15 h during the week preceding the blood sample, no sporting activity, and for a BMI of 25 (**A**), or an age of 50 years old (**B**).

The multivariable linear regression model included 12 variables, which were the 11 variables retained from the bivariate analyses plus the seasonality. The intercept for each month showed that participants whose serum was sampled in March had vitamin D values on average 27 nmol/L lower than those whose serum was sampled in August. Regarding other variables, being a man, current smoker, unemployed, having a dark skin phototype, no vitamin D supplementation in the last 12 months, no significant exposure during holidays over the last 12 months, a lower cumulative sun exposure over the last week, no sporting activities (either outdoor or indoor), and living in the North were significantly associated with a lower vitamin concentration. The vitamin D concentration was also significantly associated with age and BMI, but these associations were not linear (Table 3). The vitamin D concentration tended to decrease with age until 40–50 years and to be higher for older age classes. It tended to be maximal for BMI values around 20 and lower for values below and above 20 (Figure 3). Participants with dark skin (type V and VI) had serum vitamin D concentration 15.37 nmol/L lower than participants with light-colored skin (type I to III), and participants with tanned skin (type IV) had serum vitamin D concentration 1.95 nmol/L lower than participants with light-colored skin (type I to III). Smokers had vitamin D concentration on average 3.75 nmol/L lower than non-smokers, and unemployed participants had vitamin D concentration 2.02 nmol/L lower than participants who were either employed or retired. Participants who had no significant sun exposure during holidays over the preceding 12 months had vitamin D concentration 8.01 nmol/L lower than participants who had, and participants who practiced any sport had vitamin D concentration on average 3.89 nmol/L higher than those who did not (Table 3). Overall, the final model explained 28% of the total variance in vitamin D concentration (0.282, 95% CI (0.238; 0.324)).

### 3.4. Model Performance

The AUC of the final model was estimated at 0.77 (95% CI (0.75; 0.80)) for identifying participants with a vitamin D deficiency and at 0.82 (95% CI (0.78; 0.85)) for identifying participants with severe vitamin D deficiency. The model identified severe vitamin D deficiency with a sensitivity of 77.9% (95% CI (69.1; 85.7)), a specificity of 68.3% (95% CI (64.8; 71.9)), a positive likelihood ratio (LR+) of 2.5, and a negative likelihood ratio (LR−) of 0.3. It identified vitamin D deficiency with a sensitivity of 56.7% (95% CI (52.0; 61.8)), a specificity of 81.0% (95% CI (77.2; 84.8)), an LR+ of 3.0, and an LR− of 0.5 (Table 4).

## 4. Discussion

In the present study, barely one-fifth of the study population had sufficient vitamin D concentration, most of them had vitamin D deficiency, and almost one-tenth of the population had severe vitamin D deficiency. These results are in line with those of the last French national public health survey ESTEBAN, which was conducted between 2014 and 2015 [34].

Using data collected specifically for this purpose with a comprehensive approach taking into account most types of factors that influence serum vitamin D concentration, and using detailed information regarding sun exposure, we developed a predictive model for vitamin D concentration. The linear regression analysis identified 12 variables as independent and statistically significant predictors for vitamin D concentration, i.e., month of blood sampling, age, sex, BMI, skin phototype, employment status, smoking status, latitude, vitamin D supplementation in the last 12 months, significant exposure during holidays over the last 12 months, sun exposure in the last week, and sport practice. These predictors had various association strengths with the vitamin D concentration: the month of blood sampling was the strongest predictor, illustrating the seasonality of the vitamin D concentration, the lowest concentrations were observed at the end of winter in March, and the highest concentrations were observed in August, which is consistent with a 2008 study by Holick et al. [35]. In most published predictive models, seasonality was taken into account using four modalities corresponding to the four seasons [10,12,17,18,19]. In our model, we accounted for the cyclic shape of the seasonality, which allowed a more accurate adjustment of the regression model for the month of blood sampling.

Skin phototype was the second most strongly associated factor in the multivariable model, especially dark skin (types V and VI according to the Fitzpatrick classification), which was associated with lower serum vitamin D concentration than light-colored skin (types I to III). Surprisingly, this factor has rarely been included in previously published models [19,36], although the physiological association between skin phototype and vitamin D synthesis has been demonstrated for a long time [37]. Although sun exposure is an important significant contributor to the predictive ability of the vitamin D status models, individual sun exposure behavior had been precisely measured in only about half of the published models so far [38]. In addition, individual sun exposure has been measured in various ways regarding intensity, chronology, and surface exposed. With these limitations, these models have suggested that the lengths of time spent under the sunlight, outdoor, or tanning in the past 12 months were indeed significant contributors to the predictive ability [10,15,16,17,19,36]. To find the best measure of individual sun exposure, a section was integrated herein with detailed and various questions about sun exposure habits, actual exposure in the preceding year, and recent exposure in the preceding week. The sun exposure measures that were both the best predictors of serum 25(OH)D concentration and the easiest to collect were “significant exposure during holidays over the last 12 months” (defined as having exposed one’s bust during at least one of the holiday periods over the last 12 months) and “sun exposure last week”, and this was particularly obvious after adjustment for latitude of the place of residence and skin phototype. Moreover, the latitude of the place of residence was a significant predictor, independently of sun exposure, as participants residing in the South of France had serum vitamin D values 7.55 nmol/L higher than participants residing in the North of France. Thus, in countries with a large variation in latitude, it is extremely important to take this factor into account.

Old and oldest individuals (aged 65 years and more) are at a higher risk of vitamin D deficiency, and studies conducted on elderly patients have found that older age was associated with a higher prevalence of hypovitaminosis D [10,12,36]. However, most studies conducted in younger individuals have either found no association [15,18,19] or even a reverse association [17], and a non-linear association was found herein. Sex, as aging, has not been consistently associated with vitamin D concentration in the literature. As previously found in the study by Bolek-Berquist et al. [16], male sex was a significant predictor of lower serum 25(OH)D concentration herein. Female sex has also been found as a significant predictor of hypovitaminosis D in previous studies [12,17,19], while in most others [15,34,36,39,40], no difference in 25(OH)D concentrations between sexes was found. The association between elevated BMI and lower vitamin D concentration found herein was consistent with the results of all previous reports that have included this variable in their models [38]. Nevertheless, the magnitude of the effect of these three socio-demographic variables (age, sex, and BMI) on vitamin D concentrations seems limited in a general adult population. Regarding lifestyle factors, the smoking status and sport practice were significant predictors of vitamin D concentration, which is in agreement with previous models that have included them [38].

The set of predictors included in our final model explained about one-third of the total variability in vitamin D concentration. The unexplained remaining variability can be attributed to a variety of factors such as usual memory biases and errors regarding lifetime exposure to risk factors, variability in serum 25(OH)D measurements, or other unknown or unmeasurable factors such as genetic factors [41,42,43]. Nevertheless, the performance of our model was comparable to that of previously published models regarding AUCs [10,12,17,36,39], R^2^ [10,12,18,36,44], and sensitivity and specificity [16,17,19]. Furthermore, it has the advantage of relying on parameters that can be easily and rapidly obtained in routine care. Another strength is that we explored a large number of detailed information on sun exposure and kept the items that were both easier to collect (and easier for patients to answer) and mostly correlated with the outcome.

From a pragmatic and preventive point of view, we chose the threshold of predicted value of 50 nmol/L for two reasons. The first one is that vitamin D concentration determination should be prescribed as relevantly as possible i.e., when there is a high probability of deficiency. The second one is that it is important not to miss patients with severe vitamin D deficiency and thus to choose a threshold that maximizes the sensitivity of the model. Had we chosen a threshold of 25 nmol/L for instance, the model would certainly have had better specificity but would have had lower sensitivity, and a greater number of severe deficiency cases would have been undetected. Using this threshold of 50 nmol/L could allow almost 80% of severe deficiency cases and almost 60% of deficiency cases to be detected, and only 20% of tested positive patients would not actually be deficient.

The strengths of the present study included the testing of a large cohort of individuals of both sexes, over a wide range of age, over an inclusion period of more than one year, and using a uniform method for the measurement of serum vitamin D concentration. Performing an ad hoc study using a specific questionnaire allowed a more reliable and precise collection and measurement of predictive factors. Contrary to most published models [38], we reported the contribution of individual risk factors in the predictive ability of our model. Individual sun exposure was measured through a number of various questions, making it possible to select the measure that was the easiest to collect among those with equivalent predictive abilities. Finally, as the analytical performance of vitamin D assays is highly variable [45], the use of automated Diasorin Liaison XL assay herein, which is the most frequently used immunoassay and considered as highly reliable, is a strength. In addition to its good analytical performance, it shows a good overall correlation with the chromatographic LC-MS/MS method, the reference for measurement of circulating vitamin D, with a mean bias criterion <3% compared to the chromatographic method [45].

We have to mention some limitations of the present study. First, the assessment of potential risk factors for vitamin D deficiency was based on self-reported information. Nevertheless, this way of collecting data seems the most suitable for the objective of building a screening tool that can be easily used in clinical practice to identify individuals at high risk of deficiency and those at very low risk of deficiency in order to better target the assays. Second, we cannot ensure that our sample was representative of the population as it was based on outpatients seen at the hospital who may be different from the general population. Nevertheless, the vitamin D measurements obtained herein were consistent with those from the last French national public health survey ESTEBAN [34], which suggests that our results may be extrapolated to the French population. Third, the model is sensitive to seasonality and latitude, which means that the present results may be extrapolated to neighboring European countries of the same latitude but should be adapted before being used in countries with very different latitude.

## 5. Conclusions

In conclusion, the present study enabled the development, in a general adult population, of a predictive model for vitamin D concentration that allows the identification of individuals with severe vitamin D deficiency (serum 25(OH)D concentration <25 nmol/L) with a sensitivity of 78%. This model may not replace proper vitamin D concentration determination, as it weakly increases or decreases the post-test probability, as documented by the LR+ and LR−. Further research is needed to find the most appropriate way of using this model in the decision-making process of test and/or vitamin D supplementation prescription. The feasibility and the external validity of this model in primary care settings will have to be tested before developing a score to classify patients and measuring its impact in real life.

## Figures and Tables

**Table 1:** Characteristics of the study population (n = 2488).

Characteristics	Study Population N (%)
Season of blood sampling (n = 2488)	
Summer	468 (18.8%)
Fall	820 (33.0%)
Winter	669 (26.9%)
Spring	531 (21.3%)
Serum 25(OH)D concentration (nmol/L) (n = 2488)	
<25	195 (7.8%)
[25;50[	885 (35.6%)
[50;75[	971 (39.0%)
≥75	437 (17.6%)
Age (years) (n = 2488)	
[18;30]	551 (22.1%)
]30;40]	428 (17.2%)
]40;50]	565 (22.7%)
]50;60]	594 (23.9%)
]60;70]	350 (14.1%)
Sex (n = 2488)	
Male	975 (39.2%)
Female	1513 (60.8%)
Body mass index (kg/m^2^) (n = 2473)	
Underweight, <18.5	97 (3.9%)
Normal weight, [18.5;25[	1284 (51.9%)
Overweight, [25;30[	674 (27.3%)
Obese, ≥30	418 (16.9%)
Skin phototype (n = 2475)	
Light colored skin (type I to III)	1631 (65.9%)
Tanned skin (type IV)	698 (28.2%)
Dark skin (type V and VI)	146 (5.9%)
Education level (n = 2479)	
No diploma	171 (6.9%)
Technical school certificate	710 (28.6%)
High school diploma	788 (31.8%)
Postgraduate degree	810 (32.7%)
Employment status ^1^ (n = 2488)	
Unemployed	663 (26.6%)
Employed	1825 (73.4%)
Latitude of place of residence (n = 2473)	
North	228 (9.2%)
Center	1916 (77.5%)
South/Corsica	329 (13.3%)
Smoking status ^2^ (n = 2474)	
Yes	461 (18.6%)
No	2013 (81.4%)
Vitamin D supplementation in the last 12 months (n = 2488)	
Yes	321 (12.9%)
No	2167 (87.1%)
Intake of at least one vitamin D supplemented product in the last 12 months ^3^ (n = 2488)	
Yes	525 (21.1%)
No	1963 (78.9%)
Significant sun exposure during holidays over the last 12 months (n = 2488)	
Yes	958 (38.5%)
No	1520 (61.1%)
No holiday	10 (0.4%)
Sun exposure during the past week (n = 2449)	
0 h/day	109 (4.5%)
]0–0.5] h/day	468 (19.1%)
]0.5–1] h/day	534 (21.8%)
]1–2] h/day	588 (24.0%)
]2–3] h/day	277 (11.3%)
>3 h/day	473 (19.3%)
Practice of a sporting activity (n = 2488)	
Yes	1266 (50.9%)
No	1222 (49.1%)
Sporting activity duration (n = 2488)	
0 h/week	1222 (49.1%)
]0;2] h/week	365 (14.7%)
]2;4] h/week	331 (13.3%)
]4;6] h/week	225 (9.0%)
>6 h/week	345 (13.9%)
Intensity of sporting activity (n = 2483)	
No sporting activity	1222 (49.2%)
Only low-intensity sport	62 (2.5%)
At least one medium-intensity sport	528 (21.3%)
At least one high-intensity sport	671 (27.0%)

^1^ The unemployed group includes retirees, the disabled, and the unemployed. ^2^ “yes”: current smokers, “no”: never smoked or quit. ^3^ Consumption of at least one food product that contains added vitamin D (dairy products, oils, or fruit juices) in the last 12 months.

**Table 2:** Corrected R^2^ of the bivariable models adjusted to the month of the blood sampling.

Characteristics	Corrected R^2 †^	95% CI for R^2 †^	Final Model ^‡^
Age (years)	0.169	[0.129; 0.207]	*
Sex	0.166	[0.125; 0.204]	*
Body mass index (kg/m^2^)	0.193	[0.150; 0.233]	*
Skin phototype	0.187	[0.148; 0.222]	*
Education level	0.169	[0.127; 0.204]	
Employment status	0.167	[0.126; 0.204]	*
Latitude of place of residence	0.163	[0.120; 0.201]	*
Smoking	0.164	[0.122; 0.201]	*
Vitamin D supplementation in the last 12 months	0.176	[0.136; 0.212]	*
Intake of at least one vitamin D supplemented product ^††^	0.162	[0.122; 0.197]	
Significant sun exposure during holidays ^‡‡^	0.203	[0.163; 0.243]	*
Sun exposure last week	0.163	[0.122; 0.199]	*
Practice of a sporting activity	0.184	[0.138; 0.221]	*
Sporting activity duration	0.185	[0.142; 0.221]	
Intensity of sporting activity	0.189	[0.144; 0.227]	

Abbreviation: CI, confidence interval; ^†^ Median and 95% CI were obtained by bootstrap; ^‡^ * means that the variable is retained for the final multivariate model; ^††^ Over the last 12 months; ^‡‡^ Over the last 12 months.

**Table 3:** Multivariable regression model with 25(OH)D concentration as dependent variable (n = 2368).

**Characteristics**	**Intercept/(nmol/L) ^1^**	**95% CI ^3^**	***p*** **-Value**
Month of blood sampling			<0.001
January	54.88	[50.29; 59.48]	
February	52.82	[48.63; 57.01]	
March	52.33	[48.00; 56.67]	
April	54.81	[50.50; 59.11]	
May	60.31	[55.81; 64.80]	
June	68.17	[63.73; 72.61]	
July	75.83	[71.35; 80.30]	
August	79.32	[74.80; 83.85]	
September	77.50	[73.24; 81.76]	
October	72.90	[68.58; 77.21]	
November	67.58	[63.26; 71.91]	
December	62.02	[57.17; 66.87]	
**Characteristics**	**Regression Coefficients (nmol/L) ^2^**	**95% CI ^2^**	***p*** **-Value**
Female	1.73	[0.04; 3.43]	0.045
Age (per 5 years) ^4^	0.48	[0.02; 0.94]	0.043
Body mass index (per kg/m^2^) ^5^	−0.80	[−1.09; 0.50]	<0.001
Skin phototype ^6^ Tanned skin (type IV) Dark skin (type V and VI)	−1.95 −15.37	[−3.71; −0.19] [−18.80; −11.95]	0.030 <0.001
Unemployed	−2.02	[−4.18; 0.15]	0.068
Latitude ^7^ Center South/Corsica	0.927.55	[−2.01; 3.85] [4.12; 10.98]	0.537 <0.001
Smoker	−3.75	[−5.79; −1.70]	0.001
No vitamin D supplementation in the last 12 months	−8.73	[−11.15; −6.30]	<0.001
No significant exposure during holidays over the last 12 months	−8.01	[−9.65; −6.38]	<0.001
Sun exposure last week (per one hour/week)	0.10	[0.04; 0.16]	0.002
Practice of a sporting activity	3.89	[2.23; 5.56]	<0.001

Abbreviations: CI, confidence interval; 25(OH)D, 25-hydroxyvitamin D; BMI, body mass index; ^1^ The values regarding the month of blood sampling are the intercepts, which correspond to the mean value of serum vitamin D each month of blood sampling for the reference population; ^2^ The values corresponding to other variables of the model are regression coefficients and correspond to the change in serum vitamin D compared to the reference for each variable; ^3^ 95% CI obtained by bootstrap; ^4^ Values indicated correspond to the linear term; for the quadratic term, the values were −0.45, 95% CI [0.22; 0.67], *p* < 0.001; ^5^ Values indicated correspond to the linear term; for the quadratic term, the values were −0.40, 95% CI [−0.81; 0.00], *p* < 0.001; for the cubic term, the values were 0.48, 95% CI [0.03; 0.92], *p* < 0.001; ^6^ The reference for skin phototype is light-colored skin (type I to III); ^7^ The reference for latitude of place of residence is North.

**Table 4:** Performance of the model to predict vitamin D deficiency and severe vitamin D deficiency with a threshold of 50 nmol/L.

Vitamin D Deficiency	Parameter	Bootstrap Estimate ^1^	95% CI ^1^
Severe vitamin D deficiency	Sensitivity	77.9	[69.1; 85.7]
	Specificity	68.3	[64.8; 71.9]
Vitamin D deficiency	Sensitivity	56.7	[52.0; 61.8]
	Specificity	81.0	[77.2; 84.8]

Abbreviations: CI, confidence interval; ^1^ Bootstrap estimates based on 1000 replicates.

## Data Availability

Data described in the manuscript, code book, and analytic code will be made available upon reasonable request pending application and approval.

## Appendix A. Risk Factors for Vitamin D Deficiency Included in the Questionnaire

**Table A1:** Risk Factors for Vitamin D Deficiency Included in the Questionnaire.

Section	Items
Socio-demographic data	Country of birth Education level Residential location Type and level of employment
Clinical data	Weight Size Skin type For women: number of pregnancies, age at first pregnancy, year of birth of last child, menopausal status Smoking status Physical activities (type, number of hours) Chronic muscle, joint, or bone pain with no known cause (presence, level of intensity)
Sun exposure	Usual sun exposure during working hours: frequency and number of hours spent for outdoor work and outdoor lunch. Usual sun exposure during leisure time: number of hours spend outdoors during the week and the weekend, according to the season Sun exposure last week: number of hours Holiday sun exposure in the last 12 months: vacation spots, time spent outdoors
Treatments	Vitamin D intake in drops, ampoules, or tablets in the last 12 months: dates and dosage Dietary supplements intake over the last 12 months Taking diuretics, oral contraceptives, menopausal hormone therapy, or other treatments
Dietary intake	Intake of fish, eggs, and dairy products Intake of vitamin D supplemented foods
Exposure to artificial ultraviolet radiation	Frequency over the last 12 months
